# FMRP Associates with Cytoplasmic Granules at the Onset of Meiosis in the Human Oocyte

**DOI:** 10.1371/journal.pone.0163987

**Published:** 2016-10-03

**Authors:** Roseanne Rosario, Panagiotis Filis, Victoria Tessyman, Hazel Kinnell, Andrew J. Childs, Nicola K. Gray, Richard A. Anderson

**Affiliations:** MRC Centre for Reproductive Health, Queen’s Medical Research Institute, University of Edinburgh, 47 Little France Crescent, Edinburgh, EH16 4TJ, United Kingdom; Centre National de la Recherche Scientifique, FRANCE

## Abstract

Germ cell development and primordial follicle formation during fetal life is critical in establishing the pool of oocytes that subsequently determines the reproductive lifespan of women. Fragile X-associated primary ovarian insufficiency (FXPOI) is caused by inheritance of the *FMR1* premutation allele and approximately 20% of women with the premutation allele develop ovarian dysfunction and premature ovarian insufficiency. However, the underlying disease mechanism remains obscure, and a potential role of FMRP in human ovarian development has not been explored. We have characterised the expression of *FMR1* and FMRP in the human fetal ovary at the time of germ cell entry into meiosis through to primordial follicle formation. FMRP expression is exclusively in germ cells in the human fetal ovary. Increased FMRP expression in germ cells coincides with the loss of pluripotency-associated protein expression, and entry into meiosis is associated with FMRP granulation. In addition, we have uncovered FMRP association with components of P-bodies and stress granules, suggesting it may have a role in mRNA metabolism at the time of onset of meiosis. Therefore, this data support the hypothesis that FMRP plays a role regulating mRNAs during pivotal maturational processes in fetal germ cells, and ovarian dysfunction resulting from *FMR1* premutation may have its origins during these stages of oocyte development.

## Introduction

Female reproductive senescence largely results from the depletion of a finite ovarian follicle pool that is established during fetal life. In humans, these follicles are produced from primordial germ cells, which migrate to the gonadal ridge by the 5^th^ week of gestation, proliferate and develop into oocytes [1]. The transition from primordial germ cells to oocytes occurs from the 11^th^ week of gestation and is marked by a loss of pluripotency markers including OCT4 and LIN28 [2], incomplete cytokinesis with formation of germ cell nests and entry into meiosis [3, 4]. Oocytes progress through the first few stages of meiotic prophase I before arresting at diplotene, and following germ cell nest breakdown, are subsequently enclosed by a layer of pregranulosa cells, thus forming primordial follicles, from around 17 weeks gestation onwards [5]. Oocyte development in the human fetal ovary is relatively asynchronous, with a developmental gradient forming: pre-meiotic oogonia mainly reside in the more superficial ovarian cortex while differentiated oocytes and newly formed primordial follicles are located towards the ovarian medulla [6]. These follicles are then activated into the growth phase over the reproductive lifespan, culminating in their depletion which signals the menopause.

Premature (also termed primary) ovarian insufficiency (POI) is defined as the cessation of menses with evidence of follicle depletion before the age of 40, and has both environmental and genetic causes (reviewed in [7]). One such genetic cause is the expansion of a CGG trinucleotide repeat in the 5’ untranslated region (UTR) of the *FMR1* gene, resulting in the disorder known as fragile X-associated primary ovarian insufficiency (FXPOI) [8]. The *FMR1* allele is susceptible to expansion during germline transmission [9]; a full mutation (defined as >200 CGG repeats) of *FMR1* leads to an absence or deficiency of the FMR1 protein (FMRP) through epigenetic silencing of the gene, and is the prime cause of fragile X syndrome, while a premutation (55–200 CGG repeats) is characterised by elevated *FMR1* transcript expression [10]. Approximately 20% of women who carry the premutation allele have FXPOI [11, 12]. Prior to the onset of POI, premutation carriers have abnormal ovarian reserve biomarkers and a reduced response to controlled ovarian stimulation [13], indicating a depleted follicle pool.

The origins and onset of ovarian dysfunction in women with FXPOI are unknown, and no extended expression analysis of FMRP has been performed in the human fetal ovary. However, FMRP expression has been reported in ovarian germ cells of a 19 week old fetus carrying the full mutation and a miscarried 24 week old human fetus without fragile X syndrome; from neonatal to adult the protein has only been observed in granulosa cells [14, 15]. In mice, FMRP is present in both oocytes and granulosa cells of adult ovaries [16–18]. Two knock-in mouse models for the *FMR1* premutation have demonstrated that an intermediate length of CGG repeats is sufficient to impair female fertility [16, 19]. These mice showed an increased rate of follicular atresia indicating increased follicle activation and loss, and exhibit increased and decreased serum concentrations of FSH and 17β-estradiol respectively, which reflect the hormonal changes seen in human POI patients. *Fmr1* null mice have enlarged ovaries and exhibit precocious follicular activation [20]. Although *Fmr1* mRNA is expressed in the fetal ovary, mice with the premutation are reported to have a normal primordial follicle pool, though this is depleted more quickly than in wildtype mice, leading to ovarian insufficiency. Whether this is due to an intrinsic abnormality within the oocytes or pre-granulosa cells of primordial follicles, or in the pathways that control follicle activation is unclear, as is its possible relevance to human primordial follicle formation and oocyte development [21].

FMRP is an RNA binding protein that regulates mRNA translation as well as the transport and stability of its mRNA targets in neurons [17, 22]. In these cells, FMRP is a component of several different mRNA ribonucleotide particles (mRNP) containing granules: namely, stress granules [23], processing bodies (P-bodies) [24] and neuronal granules [25]. These granules are thought to play key roles in mRNA storage, turnover and localisation. A fourth type of RNA granule, the germ cell granule, is an evolutionary conserved feature of germ cell cytoplasm and critical for gametogenesis [26]. Within these granules, target mRNAs are associated with RNA binding proteins that control whether mRNAs are stored, translated and/or degraded [27]. This is particularly important for growing oocytes in mammals, as *de novo* synthesis of transcripts ceases as they enter meiosis, and subsequent changes in protein production is highly dependent on regulated translation of stored mRNAs [28–30].

As germ cell development and primordial follicle formation are critical in establishing lifelong reproductive potential, and the potential importance of FMRP in this process, we have explored the expression of *FMR1* mRNA and of its two paralogues *FXR1* and *FXR2*, and FMRP in the human fetal ovary at these developmental stages. By characterising the expression and localisation of FMRP in developing germ cells, and its relationship to markers of these various granule types, we hoped to gain insight into how the *FMR1* premutation may affect human oocyte development and/or folliculogenesis.

## Results

### Expression of *FMR1* and FMRP increases during human fetal ovarian development

**Fig 1:**
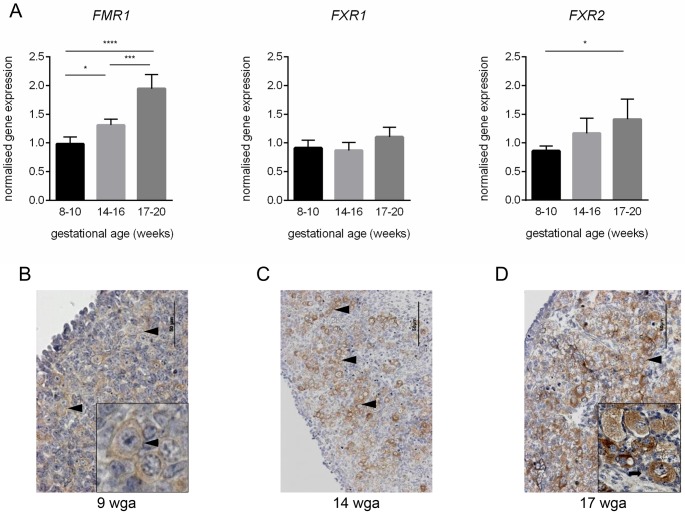
Expression of *FMR1*, *FXR1*, *FXR2* and FMRP in human fetal ovaries. A. mean ovarian mRNA levels of *FMR1* and its paralogues *FXR1* and *FXR2* among 8–10 (*n* = 5), 14–16 (*n* = 5) and 17–20 (*n* = 5) weeks of gestation. Error bars in histograms represent ±SEM. B-D. Immunohistochemical localisation of FMRP in human fetal ovaries at 9, 14 and 17 wga. FMRP is expressed in the cytoplasm of germ cells at all gestations examined, with its expression maintained in newly formed primordial follicles (D, insert). Black arrowheads indicate germ cell nests. Black arrow indicates primordial follicle. Scale bars: 50μm

Expression of *FMR1* and its two paralogues *FXR1* and *FXR2* was assessed in 8–11 weeks gestational age (wga), 14-16wga and 18-20wga human fetal ovaries using RT-qPCR, as these stages encompass key stages of germ cell development, specifically primordial germ cell proliferation, entry into meiosis and initiation of primordial follicle formation, respectively. *FMR1* transcript levels increased between 8-11wga and 14-16wga (p<0.05) and this expression continued to increase at 18-20wga (p<0.0001; Fig 1A). This change in expression across gestation was also shown for *FXR2*, however transcript levels were only significantly different between 8-11wga and 18-20wga (p<0.05). No changes were seen in the expression of *FXR1* across development. Examination of FMRP expression using immunohistochemistry revealed abundant staining in 14wga and 17wga tissue compared to 9wga tissue (Fig 1B–1D), consistent with increasing *FMR1* transcript levels during this period. FMRP expression was observed exclusively in the germ cell cytoplasm across the range of gestations examined and was maintained after germ cell nest breakdown and follicle formation (Fig 1D insert). No FMRP expression was observed in granulosa or other ovarian somatic cells, or the negative control (data not shown).

### Changes in FMRP expression and distribution coincides with the developmental progression of primordial germ cells

**Fig 2:**
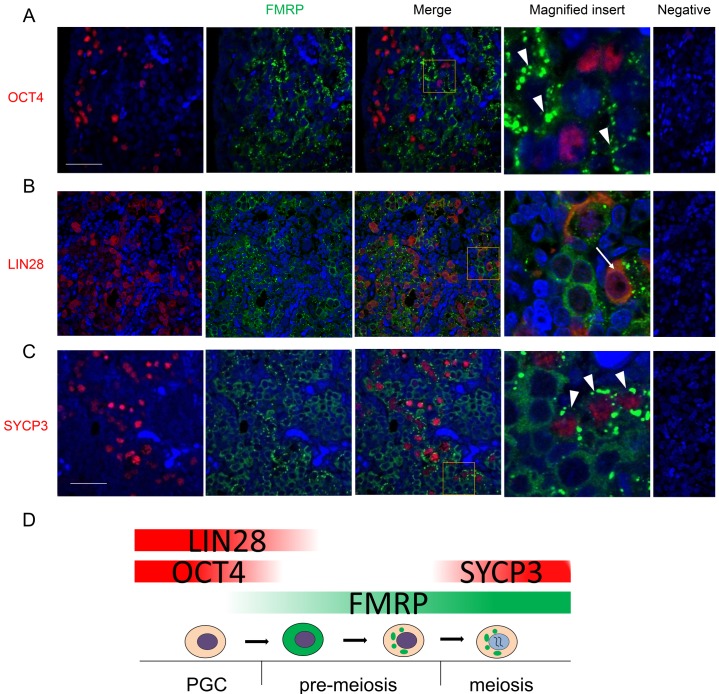
Immunohistochemical co-localisation of FMRP and primordial germ cell or meiosis markers in fetal ovaries at 14-16wga. A. FMRP and the pluripotency marker OCT4 are expressed in distinct germ cell subpopulations with only few germ cells co-expressing OCT4 and FMRP. OCT4-positive cells are located closer to the cortex and FMRP-positive cells are closer to the medulla. Note the granular distribution of FMRP (white arrowheads). B. As with OCT4, there is limited overlap between FMRP and the primordial germ cell-specific marker LIN28. Note that in germ cells co-expressing FMRP and LIN28, FMRP distribution is not granular (white arrow). C. Expression of the meiosis marker SYCP3 correlates with FMRP granulation (white arrowheads) in most but all of germ cells, suggesting that FMRP granulation precedes SYCP3 expression. D. Diagram outlining FMRP expression and granulation in germ cell development from primordial germ cells (PGCs) to meiotic oocytes. Abbreviations: ct, cortex; md, medulla. Scale bars: 50μm. Pictures are representatives from at least five different ovaries.

Double immunofluorescence was used to further characterise FMRP expression in relation to germ cell development from mitotic proliferation through to entry into meiosis. There was limited overlap between the expression of primordial germ cell/pluripotency-associated markers OCT4 or LIN28 with FMRP (Fig 2A and 2B), indicating little FMRP expression in premeiotic germ cells. At 14–16 wga, approximately 17% of OCT4 positive cells and 9% of LIN28 cells also expressed FMRP, and in these instances FMRP staining was weak and diffuse. Consistent with increased expression in more mature germ cells, FMRP-expressing germ cells were predominantly located towards the medulla of the fetal ovary. This was confirmed by co-expression of FMRP with the meiotic marker SYCP3 (Fig 2C). Strikingly, the cytoplasmic distribution of FMRP changed from diffuse to granular in SYCP3 positive germ cells. Less than 0.1% of SYCP3 positive cells had diffuse FMRP staining. Therefore, the developmental transition from primordial germ cells expressing OCT4 and/or LIN28 to meiotic germ cells coincided with increased expression of FMRP and formation of FMRP-rich foci (Fig 2D).

### FMRP granules in fetal ovary show variable degrees of co-localisation with stress granule and P-body markers

**Fig 3:**
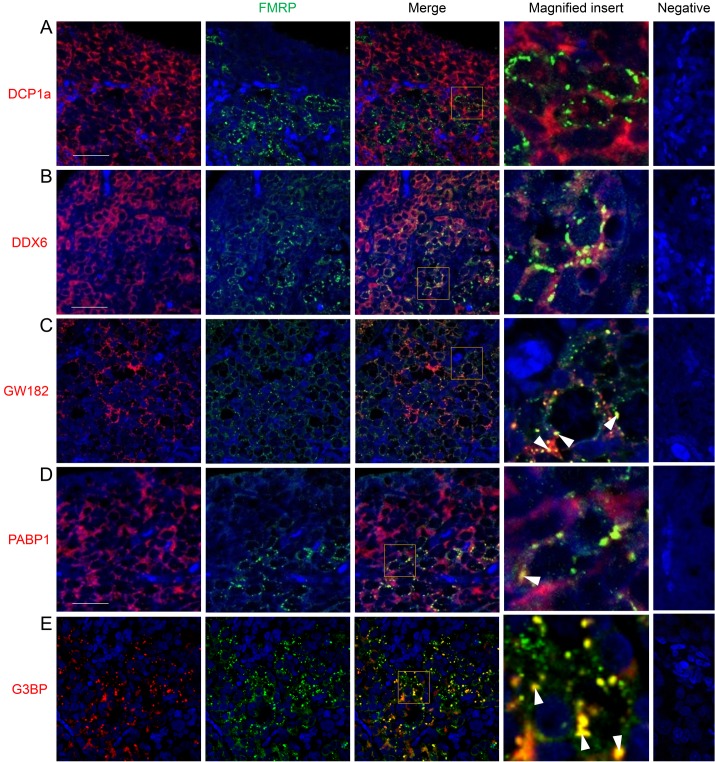
Immunohistochemical co-localisation of FMRP and P-body or stress granule markers in 2^nd^ trimester human fetal ovaries. Yellow signal indicates co-localisation. A. FMRP and the P-body marker DCP1a are expressed in distinct compartments within germ cells and no expression overlap is evident. B. The distribution of the P-body marker DDX6 is generally diffuse; some degree of co-localisation between FMRP granules and DDX6 is evident, possibly the result of random association of DDX6 with FMRP. C. The distribution of the P-body marker GW182 is distinctly granular in the human fetal ovary and there is a degree of association of GW182 and FMRP granules (white arrowheads). D. The distribution of the stress granule marker PABP1 is more diffuse towards the cortex and more granular towards the medulla; as with GW182, some PABP1 granules co-localise with FMRP granules. E. The distribution of the stress granule marker G3BP is distinctly granular. A portion of FMRP granules co-localise with G3BP Abbreviations: ct, cortex; md, medulla. Scale bars: 50μm. Pictures are representatives from at least five different ovaries.

Given that FMRP could be localised to a range of mRNP granules in somatic cells, we attempted to directly visualise RNA granules by *in situ* hybridisation, but this did not provide the necessary resolution (data not shown). Therefore, the FMRP-rich foci observed in human fetal germ cells were further characterised with double immunofluorescence using an array of known stress granule or P-body protein components. Although these granules are distinct and can be identified by specific markers, they are also dynamic and share some mRNA and protein components [31]. The P-body marker DCP1a was diffuse in the cytoplasm of both germ and somatic cells and its distribution did not overlap with FMRP (Fig 3A). DDX6, another P-body marker, was only found in germs cells. There was limited overlap between FMRP granules and DDX6, which may reflect the diffuse nature of the DDX6 staining (Fig 3B). Similarly, partial overlap in expression with FMRP granules was observed with the stress granule marker PABP1, as its expression was detected in the germ cell cytoplasm where its staining too was diffuse (Fig 3D). In contrast, the P-body marker GW182 was distinctly granular in the majority of germ cells and co-localised with a small proportion of FMRP granules (Fig 3C), as did that of G3BP, another stress granule marker, which also demonstrated granular expression in the germ cell cytoplasm (Fig 3E).

**Fig 4:**
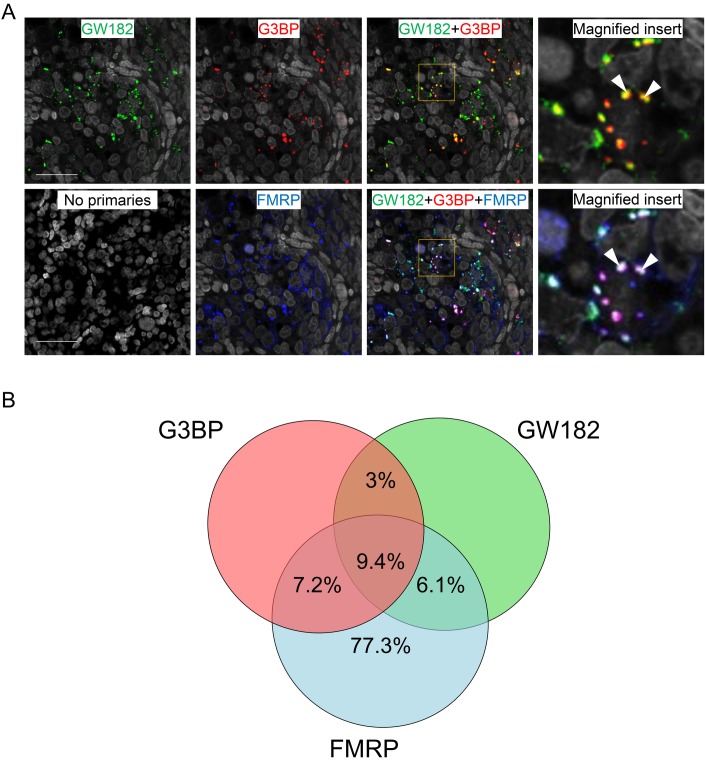
Immunohistochemical co-localisation of FMRP, GW182 and G3BP in human fetal ovaries at 15 wga. A. Yellow signal indicates co-expression of G3BP and GW182. Cyan signal indicates co-expression of GW182 and FMRP. Magenta signal indicates co-expression of G3BP and FMRP. White signal indicates co-expression of G3BP, GW182 and FMRP. B. Venn diagram depicting the relationship between granule components. The majority of granules positive for both G3BP and GW182 contained FMRP (75.5%).

Triple immunofluorescence against FMRP, GW182 and G3BP was therefore performed to investigate further the distribution of FMRP in foci containing P-body and stress granules markers (Fig 4A). The relationship between granule components is depicted in Fig 4B. 16.6% and 15.5% of FMRP foci co-expressed either G3BP or GW182, respectively, while 9.4% of FMRP granules co-localised with both granule components. GW182 granules showed a limited degree of co-localisation with G3BP granules (Fig 4B) and in those instances, FMRP was also present in a majority (75.5%), suggesting that FMRP may not only be a dynamic component of a subset of P-body and S-granules but may also be exchanged between them or play a role in promoting their fusion.

## Discussion

Establishment of the primordial follicle pool during fetal life is key in underpinning women’s reproductive potential. Factors affecting both the quality and quantity of the ovarian reserve may significantly impact a woman’s fertility and her ability to conceive. Premutation of *FMR1* is implicated in human FXPOI, and female transgenic mice harbouring the premutation allele have impaired fertility [16, 19]. However, the timing of onset of ovarian dysfunction in FXPOI, and the stage(s) of follicle development it perturbs are unknown. We therefore sought to extend the present knowledge of FMRP expression to an earlier stage of ovarian development, thus providing insight into how disruption of FMRP expression prior to, or during the establishment of the ovarian reserve may contribute to FXPOI.

We studied the expression of *FMR1*, and its protein product FMRP, in the ovaries of human fetuses, from primordial germ cell proliferation through onset of meiosis to primordial follicle formation. FMRP expression was widespread in oocytes in ovaries at 14–17 weeks gestation, including in those within newly formed primordial follicles at the latest gestations examined. The increase in both *FMR1* mRNA and FMRP across gestation, the specific expression of FMRP in germ cells, and the marked change in expression within germ cells at the onset of meiosis suggest that FMRP is associated with processes relating to germ cell differentiation at this critical stage of human ovarian development. Furthermore, the specific localisation of FMRP to the germ cell cytoplasm is consistent with roles in mRNA translation/stability/localisation. We also demonstrated expression of the *FMR1* paralogues *FXR1* and *FXR2*, the former of which also showed increasing expression with gestation; a pattern not seen for *FXR2*. The proteins expressed by *FXR1* and *FXR2* show variable co-localisation with FMRP [32], and are also thought to be involved in RNA binding and processing.

The primary function of FMRP is to repress the translation of specific RNAs. Indeed, in neuronal tissues the majority of FMRP is found in complexes that contain stalled ribosomes in addition to its target RNAs [33]. In support of a role for FMRP in regulating translation in the human fetal ovary, we identified a novel, stage-specific relocalisation of FMRP to distinct cytoplasmic granules during germ cell differentiation. We found FMRP to be diffusely distributed in the cytoplasm of premeiotic germ cells (identified by OCT4 and LIN28 expression), but observed a redistribution of the protein to distinct granules in meiotic germ cells (indicated by SYCP3 expression). As no SYCP3-positive germ cells with diffuse FMRP were observed, but SYCP3-negative germ cells with granular FMRP were evident, it seems likely that the transition from diffuse to granular FMRP occurs before or during the initiation of meiosis.

The composition of these FMRP-containing granules was further characterised to provide initial insight into the biological pathways they might participate in during germ cell maturation. Of the markers employed, G3BP and GW182 were the most informative and showed varied degrees of co-localisation with FMRP granules. G3BP is typically found in stress granules, which are thought to act as repositories for mRNA storage [34]. GW182 is observed in P-bodies, which are associated with miRNA-mediated gene silencing pathways that culminate in both translational repression and mRNA degradation [35, 36]. Given that GW182-mediated mRNA decay requires DCP1 [37], whose expression we found did not overlap with FMRP (Fig 3A), it seems likely that GW182-containing FMRP granules are not sites of miRNA-mediated degradation but rather foci of miRNA-mediated translational repression. The appearance of these granules at the onset of meiosis raises the possibility that FMRP is repressing mRNAs whose translation must be silenced to allow the mitosis-meiosis transition. Moreover, the observation of granules that contained both G3BP and GW182 may reflect the dynamic nature of stress granules and P-bodies, which can physically interact with each other, leading to the speculation that repressed FMRP-bound mRNAs can be transferred from stress granules to P-bodies for storage or degradation [31, 38]. As FMRP was found in the majority of granules containing both G3BP and GW182, it may have a role in the association of stress granules and P-bodies in human ovarian germ cells or even the co-ordination of the movement of some of its target mRNAs between these granules, as it does in hippocampal neurons [39]. Determining the functional significance of these granules in meiosis will require the identification of relevant target mRNAs, as no RNA targets of FMRP in the fetal ovary have been described.

The localisation of FMRP granules to germ cell cytoplasm leads to the hypothesis that these structures are in fact, a class of germ cell granule, as these share many components with stress granules and P-bodies, including GW182 [40]. Loss of RNAs found in these granules causes failure of primordial germ cell migration, reduced germ cell proliferation, pre-meiotic germ cell death, and meiotic defects [41–46]. However the molecular mechanisms that underlie germ cell granule involvement in these phenotypes is unclear and mammalian germ cell granules remain poorly understood.

Translational control of mRNA plays a central role in regulating gene expression during oogenesis: during their growth phase, mammalian oocytes are transcriptionally active but mRNA synthesis drops to very low or undetectable levels at the onset of meiosis [47]. Loss of this translational control, which may occur in FXPOI patients, may have more global effects and secondary consequences through the dysregulation of primary mRNA targets of FMRP. These results provide a basis for this occuring during fetal life, with consequences for the quality and quantity of the follicular reserve.

In conclusion, the changes in FMR1 mRNA expression and FMRP localisation at the time of transition from mitosis to meiosis in the human fetal ovary, suggests that FMRP is associated with important maturational processes in germ cells, and dysfunction resulting from *FMR1* premutation may have its origins at this stage of oocyte development. The biological function of FMRP granules in human fetal oocytes is unclear, yet since they associate with components of P-bodies and stress granules it is likely that they are involved in mRNA metabolism at the time of onset of meiosis, such as the repression and/or destruction of mRNAs required for the mitosis-meiosis transition.

## Materials and Methods

### Ethics statement

Ethical approval for this study was obtained from Lothian Research Ethics Committee (study code LREC 08/S1101/1) which oversees research in Edinburgh, and women gave informed written consent.

### Collection of human fetal ovaries

Human fetuses (8–20 weeks gestational age (wga)) were obtained directly from patients at local hospitals after elective termination of pregnancy, and all fetuses used in this study were morphologically normal. Gestational age was determined by ultrasound scan, and confirmed (for second trimester fetuses) by direct measurement of foot length. The sex of first trimester fetal gonads was determined by PCR for the SRY gene [48]. Extra-ovarian tissue was removed from dissected ovaries, which were then either snap frozen on dry ice and stored at -80°C for subsequent RNA extraction, or fixed in Bouins or 4% neutral buffered formalin (NBF) for 2–3 hours before processing into paraffin blocks for immunohistochemical analysis.

### RNA extraction, cDNA synthesis and RT-qPCR

RNA was extracted with the RNeasy Micro Kit (Qiagen, Crawley, UK; Cat #74004). cDNA was prepared using the Maxima^®^ First Strand cDNA Synthesis Kit (Fermentas, Loughborough, UK; #K1642) according to the manufacturer’s specifications. RT-qPCRs were performed using the Brilliant III Ultra-Fast QRT-PCR (Agilent Technologies, Stockport, UK; #600880) in 10μl volumes according to the manufacturer’s instructions. Primers are outlined in Table 1. Primer pair efficiencies were calculated with the LinReg PCR applet [49]. Each reaction was performed in a final volume of 10 μL, with 1x Brilliant III SYBR Green qPCR Master Mix (Agilent Technologies, Santa Clara, CA, USA), 20pmol of each primer and 2 μL of diluted cDNA. Each cDNA sample was analysed in triplicate. Target genes were normalised to the expression of *RPL32*. Data analysis for relative quantification of gene expression and calculation of standard deviations was performed as outlined by [50].

**Table 1:** Sequences of forward and reverse primers for real time PCR.

Gene	Forward	Reverse
*FMR1*	CAGGGCTGAAGAGAAGATGG	ACAGGAGGTGGGAATCTGA
*FXR1*	GGTTGGCTAAAGTTCGGATG	TAGCACACGCCTCTCTCAAA
*FXR2*	AGGGGATGAAGTGGAGGTTT	GAAGAAGCTGCCTTTGGTTG
*RPL32*	CATCTCCTTCTCGGCATCA	AACCCTGTTGTCAATGCCTC

### Immunohistochemistry

All antibodies, visualisation dyes, their dilutions and source information are given in Table 2. 5 μm thick fetal ovary tissue sections were de-waxed and antigen retrieval was performed in 10mM sodium citrate buffer using pressure cooker (125°C for 30s, then 90°C for 10s). In each case, peroxidase block was performed using DAKO REAL Peroxidase block (DAKO, Cambridge, UK; #5203) for 10min, followed by a biotin/streptavidin block (Vector Laboratories, Peterborough, UK; #SP2002) according to the manufacturer’s guidelines.

**Table 2:** Primary antibodies, secondary antibodies and fluorescent dyes used.

Primary Antibodies	Dilutions		Catalogue #	Company
	*DAB*	*fluorescence*		
rabbit anti-FMRP	1 in 2000	1 in 250	ab17722	Abcam, Cambridge, UK
mouse anti-OCT4		1 in 700	sc-5279	Santa Cruz Biotechnology, SantaCruz, USA
rabbit anti-LIN28		1 in 10,000	ab46020	Abcam Cambridge, UK
rabbit anti-SYCP3		1 in 50,000	ab150292	Abcam Cambridge, UK
rabbit anti-DCP1a		1 in 6,000	ab47811	Abcam Cambridge, UK
rabbit anti-DDX6		1 in 8,000	A300-461A	Cambridge Bioscience, Cambridge, UK
mouse anti-G3BP		1 in 16,000	611126	BD Biosciences, Erembodegem, Belgium
mouse anti-GW182		1 in 500	sc-56314	Santa Cruz Biotechnology, Santa Cruz, USA
rabbit anti-PABP1		1 in 6,000	N/A	from Prof. Nicola Gray
Secondary Antibodies				
biotinylated goat anti-rabbit	1 in 500	1 in 500	E0432	Dako, Cambridge, UK
biotinylated goat anti-mouse		1 in 500	BA-9200	Vector, Peterborough, UK
Peroxidase-conjugated goat anti rabbit		1 in 500	P0448	Dako, Cambridge, UK
Peroxidase-conjugated goat anti-mouse		1 in 500	P0447	Dako, Cambridge, UK
Streptavidin-conjugated HRP	1 in 1000		SA-5004	Vector, Peterborough, UK
Visualisation Dyes				
streptavidin-conjugated Alexa Fluor 488		1 in 500	S-11223	Life Technologies Ltd, Paisley, UK
Cy3-conjugated Tyramide		1 in 50	NEL744B001KT	Perkin Elmer, Cambridge, UK
Cy5-conjugated Tyramide		1 in 50	NEL745B001KT	Perkin Elmer, Cambridge, UK
Liquid DAB + Substrate Chromogen System	N/A		K3468	Dako, Cambridge, UK
DAPI		1 in 1000	D9542	Sigma-Aldrich, Dorset, UK

For 3,3'-Diaminobenzidine tetrahydrochloride (DAB) detection, sections were rinsed in Tris-buffered saline (TBS), blocked in 20% normal goat serum + 5% bovine serum albumin (BSA) (Sigma-Aldrich, Poole, UK) in TBS (NGS/BSA/TBS) for 30min, and incubated with anti-FMRP antibody diluted in NGS/BSA/TBS overnight. The following day sections were washed 1 x 5min in TBS + 0.05% Triton X-100 (Sigma-Aldrich), followed by 1 x 5min in TBS. The appropriate biotin-conjugated secondary antibody was diluted in NGS/BSA/TBS, applied to the section for 30min and washed as above. Streptavidin-conjugate horseradish peroxidase (HRP) was diluted in TBS and applied on the sections for 30min and again washed as above. DAB detection was then carried using the Liquid DAB + Substrate Chromogen System according to the manufacturer’s instructions. Staining was examined under a microscope and the reaction stopped by transferring to TBS when staining reached desired intensity. Sections were counterstained with haematoxylin, dehydrated and mounted using Pertex (CellPath Ltd, Newtown Powys, UK).

For double immunofluorescence, the first primary antibody used was either anti-OCT4, anti-LIN28, anti-SYCP3, anti-DCP1a, anti-DDX6, anti-GW182 or anti-PABP1, and the second primary applied was anti-FMRP. Sections were rinsed in phosphate-buffered saline (PBS), blocked in 20% goat serum + 5% BSA in PBS (NGS/BSA/PBS) for 30min and incubated with the first primary antibody diluted as indicated in Table 2 in NGS/BSA/PBS overnight. The following day sections were washed 1 x 5min in PBS + 0.05% Triton X-100, followed by 1 x 5min in PBS. The appropriate peroxidase-conjugated secondary antibody was diluted in NGS/BSA/PBS, applied to the section for 30min and washed as above. Cy3-conjugated tyramide was applied on the sections according to the manufacturer’s specifications for 10min, after which time sections were washed as above. Tissue-bound antibodies were denatured by microwaving sections in 10mM sodium citrate buffer for 4min at high-power, followed by 4min at medium power and left to cool for 20-30min. Sections were blocked, incubated overnight with the second primary (anti-FMRP) diluted in NGS/BSA/PBS and washed in the same manner as the first primary. Biotinylated Goat anti-rabbit was diluted in NGS/BSA/PBS and applied to the sections for 30min, followed by washes as above. Alexa Fluor488-streptavidin conjugate was diluted in NGS/BSA/PBS, applied to the sections for 60min and rinsed as above. Nuclei were counter stained with DAPI (diluted in PBS) for 10-20min, and sections were washed as above and mounted in Permafluor (Thermo Scientific, Asheville, North Carolina, USA). Triple immunofluorescence was conducted as above probing sections sequentially with anti-GW182, anti-G3BP and anti-FMRP primaries. Each primary was detected using Cy3-conjugated tyramide, Cy5-conjugated tyramide and Alexa Fluor488-conjugated streptavidin respectively. FMRP, G3BP and GW182 granules and their double and triple combinations were counted in a field of view of 0.05mm^2^ from three stained ovarian sections spanning 14–16 wga using ImageJ.
